# Investigating the trajectory of post-COVID impairments: a longitudinal study in Sweden

**DOI:** 10.3389/fpsyg.2024.1402750

**Published:** 2024-06-10

**Authors:** Farzaneh Badinlou, Tamar Abzhandadze, Fatemeh Rahimian, Markus Jansson-Fröjmark, Maria Hedman-Lagerlöf, Tobias Lundgren

**Affiliations:** ^1^Centre for Psychiatry Research, Department of Clinical Neuroscience, Karolinska Institute and Stockholm Health Care Services, Region Stockholm, Stockholm, Sweden; ^2^Medical Unit Allied Health Professionals, Women’s Health and Allied Health Professionals Theme, Karolinska University Hospital, Solna, Sweden; ^3^Institute of Neuroscience and Physiology, The Sahlgrenska Academy, University of Gothenburg, Gothenburg, Sweden; ^4^Department of Occupational Therapy and Physiotherapy, Sahlgrenska University Hospital, Gothenburg, Sweden; ^5^Division of Clinical Geriatrics, Department of Neurobiology, Care Sciences and Society (NVS), Karolinska Institutet, Stockholm, Sweden; ^6^Research Institutes of Sweden, Department of Computer Science, Stockholm, Sweden

**Keywords:** contextual factors, COVID-19, factors related to body functions, longitudinal study, post-COVID impairments, SARS-CoV-2

## Abstract

**Introduction:**

Individuals recovering from COVID-19 often experience a range of post-recovery symptoms. However, the literature on post-COVID-19 symptoms reveals conflicting results, necessitating a heightened focus on longitudinal studies to comprehend the trajectory of impairments over time. Our study aimed to investigate changes in long-term impairments among individuals infected with COVID-19 and explore potential predictors influencing these changes.

**Methods:**

We conducted a web-survey targeting individuals that had been infected with COVID-19 at four time-points: T0 (baseline), T1 (three months), T2 (six months), and T3 (twelve months). The survey included contextual factors, factors related to body functions and structures, and post-COVID impairments. The longitudinal sample included 213 individuals (with a mean age of 48.92 years). Linear mixed models were employed to analyze changes in post-COVID impairments over time and identify impacting factors.

**Results:**

Findings revealed a general decline in post-COVID impairments over time, with each symptom exhibiting a dynamic pattern of fluctuations. Factors such as initial infection severity, education level, and work status were significantly associated with the levels of impairments.

**Discussion:**

The study emphasizes that post-COVID impairments are not static but exhibit variations over time. Personalized care, especially for vulnerable populations, is crucial. The results underscore the need for long-term monitoring and multidisciplinary treatment approaches. Targeted support and interventions are highlighted for individuals with severe initial infections and those in socioeconomically disadvantaged groups.

## 1 Introduction

A substantial proportion of individuals affected by COVID-19, irrespective of the initial disease severity, undergo a diverse spectrum of post-infection symptoms following the acute phase of COVID-19 (Augustin et al., 2021; Lopez-Leon et al., 2021; Michelen et al., 2021; Schou et al., 2021; Bull-Otterson et al., 2022; Ceban et al., 2022; Cervia et al., 2022; Soriano et al., 2022; Badinlou et al., 2023). Persistent symptoms following COVID-19 encompass multiple-organ impairments, including respiratory function, cognitive abilities, cardiovascular health, and muscular functions (Davis et al., 2021; Dixit et al., 2021; Fajar et al., 2021; Ferrucci et al., 2021; Liu et al., 2021; Torres-Castro et al., 2021; Dennis et al., 2023). Fatigue emerges as the most frequently reported symptom (Carfì et al., 2020; Davis et al., 2021; El Sayed et al., 2021; Galal et al., 2021; Logue et al., 2021; Sudre et al., 2021; Sykes et al., 2021; Almas et al., 2022; Badinlou et al., 2023).

Longitudinal studies revealed a dynamic pattern in post-COVID symptoms over time, reflecting the complexity and variability of the disease’s long-term effects (Huang et al., 2021; Hellemons, 2022; Tran et al., 2022). This complexity is manifested in a gradual decrease over time in the prevalence of certain symptoms, such as loss of appetite, change/loss of taste, and cough. Conversely, no specific change in prevalence was observed for symptoms like word-finding problems and dyspnea, while an increase was observed in symptoms such as neck-, back-, and low back pain, as well as paresthesia (Tran et al., 2022). Additionally, there were significant decreases in fatigue or muscle weakness over time, whereas the prevalence of chest pain, myalgia, and headache displayed a significant increase over time (Huang et al., 2022). Fatigue was the most commonly reported long-term symptom of COVID-19, but its prevalence decreased significantly over time (Hellemons, 2022). Our recent study (Badinlou et al., 2023) found that many people with confirmed or probable SARS-CoV-2 infections experienced moderate-to-severe impairments from COVID-19, especially fatigue as a persistent symptom. Additionally, several factors like age, education, employment status, timing of initial infection, hospitalization, COVID-19 vaccination, and the infection’s severity during the acute phase were linked to these impairments (Badinlou et al., 2023). These findings highlight the changing patterns of post-COVID-19 symptoms, emphasizing the need for more research to comprehend and manage the long-term effects of the infection.

The current body of research offers inconclusive findings regarding the progression of long-term symptoms after recovering from COVID-19 infection, underscoring the need for more longitudinal research to determine the trajectory of post-COVID impairments. Hence, we have aimed to investigate self-reported changes in long-term impairments among individuals infected with COVID-19 over time and to explore potential predictors that may influence these changes.

## 2 Materials and methods

### 2.1 Study design and study sample

In this longitudinal and prospective study, we used data from a web-based survey that aimed to study the impact of COVID-19 infection on a cohort of Swedish individuals. We recruited participants through convenience sampling by distributing electronic posters on COVID-19-related Facebook groups, the Swedish COVID-19 Association (Svenska Covidföreningen), and the Karolinska Institutet’s website. Participants were provided access to the web-based survey through Research Electronic Data Capture (REDCap), hosted locally at Karolinska Institutet (Harris et al., 2009, 2019). Inclusion criteria were: (i) Infected with COVID-19 (comprising both suspected COVID-19 infection and confirmed COVID-19 infection supported by tests for COVID-19 virus (PCR) and/or rapid antigen test); (ii) age 18 years or older; (iii) ability to understand Swedish language. Due to the longitudinal nature of this study, outcome data at all time points was needed for the respondent to be included in the study.

Data on post-COVID impairments were collected at four time points; first time point or T0 (baseline, February/March 2022), the second time point or T1 (June/July 2022), the third time point or T2 (September/October 2022), and the fourth time point or T3 (February/March 2023). The number of participants in each cross-sectional data collection varied. In the initial measurement point of the study (T0), a total of 501 participants responded. Out of the total, 213 individuals (42.5%) completed all four surveys and were included in the longitudinal analysis (Table 1).

**Table 1:** Characteristics of the study sample (*N* = 213).

Variables	*n*	%
**Personal factors**		
Sex		
Male	22	10.3
Education level		
Lower education	56	26.3
Higher education	157	73.7
Marital status		
Single	40	18.8
Married	94	44.1
In a relationship	60	28.2
Widowed/Divorced/Separated	19	8.9
Work status		
Working full time/part time	138	64.8
Retired	13	6.1
Sick leave	51	23.9
Unemployed/unpaid work/student	11	5.1
Self-rated economic status		
Below average	29	13.6
Average	104	48.8
Above average	80	37.6
**Environmental factors**		
Vaccinated against COVID-19		
Yes	181	85
No	32	15
Hospitalization for COVID-19		
Yes	28	13.3
No	183	86.7
Treatments for post-COVID-19 complaints		
Yes	120	56.6
No	92	43.4

#### 2.1.1 Ethical approval

The study was approved by the Swedish national ethical board (dnr 2021–06617-01) and written informed consent was obtained from all participants. All procedures utilized in collecting data for the current paper follow the ethical standards of the Helsinki Declaration of 1964 and subsequent amendments.

### 2.2 Measures

#### 2.2.1 Contextual factors

The contextual factors in this study encompassed both personal and environmental factors. Personal factors included demographic aspects such as age, gender, level of education, marital status, employment status, and economic status.

Environmental factors comprised whether individuals had been vaccinated against COVID-19 (binary question yes/no), whether they received treatment for post-COVID symptoms (binary question yes/no), if they had been hospitalized due to COVID-19 (binary question yes/no), and the time of their first infection in which respondents were asked to report date of their infection/infections/positive COVID-19 test/tests (year and month). The variable was categorized into those affected for the first time in the year 2020 versus those affected for the first time in the subsequent years of 2021 and 2022. This categorization aligns with our earlier study, which demonstrated that individuals experiencing their initial infection during the first and second waves of the pandemic in Sweden (spring and autumn of 2020), encountered a greater post-COVID complaints (Badinlou et al., 2023).

#### 2.2.2 Factors related to body functions and structures

Factors related to body functions and structures encompassed belonging to a high-risk group for COVID-19 and the severity of COVID-19 infection during the acute phase. Belonging to a high-risk group for COVID-19 was evaluated using a single item (binary question yes/ no), where respondents identified if they belonged or had belonged to the high-risk group for COVID-19, including conditions such as high blood pressure, angina, stroke, heart disease, diabetes, cancer, smoking, respiratory diseases, and impaired immune system.

The severity of COVID-19 infection in the acute phase was assessed using a questionnaire consisting of 15 items used in our previous studies (Badinlou et al., 2022, 2023, 2024). Participants rated the intensity of these symptoms experienced at the onset of the infection or infections and during the subsequent 4 weeks using a 4-point scale (0 = no, 1 = mild, 2 = moderate, 3 = severe). The sum of the respondents’ ratings for the 15 COVID-19 symptom items was utilized to calculate the severity of the COVID-19 infection during the acute phase, resulting in a score range of 0 to 45.

#### 2.2.3 Outcomes

The outcomes of this study were post-COVID impairments. Data on post-COVID impairments were collected via a questionnaire, which developed and utilized in our previous studies (Badinlou et al., 2022, 2023, 2024). This questionnaire comprised 54 items, categorized into four sub-categories based on the International Classification of Functioning, Disability, and Health (World Health Organization, 2001) as impairments in mental functions including impaired orientation, brain fatigue, lack of appetite, sleep problems, concentration difficulties, attention difficulties, memory problems, impaired organization and planning, impaired mental functions of language, depression, anxiety, stress, obsessions, and compulsions; impairments in sensory functions and pain including poor quality of vision, dry/red/itchy eyes, ringing in ears or tinnitus, dizziness, disturbed balance, loss of taste, loss of smell, feeling of numbness/tingling, generalized pain, pain in head, chest pain, pain in stomach or abdomen, joint pain, and pain in multiple body parts; impairments in body system functions including voice problems, impaired heart functions, respiratory distress, cough, tiredness or lack of energy (physical), shortness of breath, sore throat/difficult to swallow, vomiting, impaired nutrient uptake, diarrhea, weight change, nausea, fever/feeling of fever, chills/the feeling of freezing, impaired sexual desire and functions, impaired mobility of movement, decreased muscle power, movement problems, skin changes, rash, and hair loss; and impairments in activities and participation including difficulty taking care of yourself, impaired control of other diseases and drugs, keep special diet, and difficulties in doing housework, impaired work ability/study ability, and difficulty participating in leisure activities (see Supplementary Table S1 for a full description of the items of post-COVID impairments). Each item rated on a 4-point Likert scale (0 = no, 1 = mild, 2 = moderate, 3 = severe). The respondents’ answers to each sub-category of post-COVID impairments were summed up and divided by the number of items to obtain means for each sub-category (Badinlou et al., 2022, 2023, 2024). We assessed the reliability of the post-COVID questionnaire across four time points using the Intraclass Correlation Coefficient (ICC). The ICC values were 0.79 for impairments in mental functions (95% Confidence Interval, CI: 0.74–0.83, *p* < 0.00), 0.80 for impairments in sensory functions and pain (95% CI: 0.76–0.84, *p* < 0.00), 0.83 for impairments in body system functions (95% CI: 0.79–0.88, *p* < 0.00), and 0.80 for impairments in activities and participation (95% CI: 0.76–0.84, *p* < 0.00). An ICC value of ≥0.70 was considered acceptable. These findings demonstrate a high level of reliability for the post-COVID questionnaire over time.

### 2.3 Statistical analysis

Descriptive statistics for contextual factors and factors related to body functions and structures are provided in terms of percentages, means, and standard deviations. The prevalence of post-COVID impairment symptoms over time—baseline (T0), 3-month (T1), 6-month (T2), and 12-month follow-up (T3)—is presented as percentages of cases. The general linear mixed models were used to assess the impact of contextual factors and factors related to body functions and structures on changes in post-COVID impairments over time. Participation in a given analysis was contingent on data availability from all four measurements for a specific post-COVID impairment outcome. The models were fitted separately for each of the post-COVID impairments as the dependent variables. Time, age, gender, level of education, marital status, employment status, economic status, COVID-19 vaccination, time of first infection, treatment for post-COVID complaints, hospitalized due to COVID-19, belonging to risk group, and severity of COVID-19 infection were entered into each of the models as fixed effects. We treated individual studies as random effects. In addition, for purposes of analysis, categorical variables were dichotomized for obtaining balanced distribution among variable categories; marital status (not in relationship/in relationship), work status (not working/working), economic status (average and below average/above average), vaccinated against COVID-19 (yes/no), and time of first infection (during year 2020/ during the year 2021 and 2022).

Statistical analysis was carried out using the IBM Statistical Software Package for Social Science (SPSS; version 26) and the R-package lme4 (Bates et al., 2015). The alpha value for two-tailed significance was set at 0.05.

## 3 Results

### 3.1 Descriptive statistics for contextual factors

There were 213 individuals (mean (SD) age 48.92 (10.82) years, range 19–81 years) who completed all four surveys and were included in the study. We examined whether various sociodemographic factors could predict participants’ completion of surveys at each time point. Our findings indicated that there were no significant differences between individuals who completed surveys at all time points and those who did not regarding gender, age, education level, marital status, work status, and economic status. The detailed characteristics of the participants are presented in Table 1.

### 3.2 Descriptive statistics for factors related to body functions and structures

Table 2 presents descriptive analysis of factors related to body functions and structures. In total, 134 (62.9%) of the participants were infected with COVID-19 for the first time during the first and second wave (spring and autumn 2020) of COVID-19 in Sweden and 46 (21.6%) of the participants were in a high-risk group for COVID-19. The severity of COVID-19 infection in the acute phase ranged between 2 and 44 (*M* = 25.1, SD = 7.5). The most common symptoms at disease onset were fatigue (99%), cognitive difficulties (91.6%), headache/migraine (91.2%), and aches or pain in body (88.5%).

**Table 2:** Descriptive statistics of factors related to body functions and structures.

Variables	*n*	%
Infected with COVID-19 for the first time		
First and second wave of COVID-19 in Sweden, spring and autumn 2020	134	62.9
During the year 2021 and 2022	74	34.7
Belonging to a high-risk group for COVID-19		
Yes	46	21.8
No	165	78.2

### 3.3 Post-COVID impairments

Table 3 presents descriptive analysis of post-COVID impairments over time in the longitudinal sample. The results suggested a general trend of decrease in impairments over the initial time points, with a slight increase or stabilization toward the end (see Supplementary Table S1 for full descriptive analysis). The relatively high Intraclass Correlation Coefficient (ICC) values across all categories indicates good reliability in these measurements over the different time points.

**Table 3:** Descriptive statistics of post-COVID impairments at the four time points (*N* = 213).

Outcomes	T0		T1		T2		T3		ICC
	Mean	SD	Mean	SD	Mean	SD	Mean	SD	
Impairments in mental functions	1.30	0.64	1.27	0.67	1.23	0.65	1.25	0.65	0.79
Impairments in sensory functions and pain	1.15	0.62	1.04	0.65	1.01	0.68	1.05	0.68	0.80
Impairments in body system functions	1.06	0.55	0.91	0.56	0.92	0.59	0.97	0.59	0.83
Impairments in activities and participation	1.47	0.74	1.34	0.81	1.31	0.83	1.39	0.84	0.80

ICC, Intraclass Correlation Coefficient. Baseline (T0), 3-month (T1), 6-month (T2), and 12-month follow-up (T3).

Concerning impairments in mental function, our results indicated various trends for different mental health and cognitive issues over time. Some symptoms like impaired language functions and sleep issues showed a deterioration, others like memory and attention problems showed slight improvements or stability, and the rest, like depression and stress, exhibited fluctuating patterns (Table 4).

**Table 4:** Prevalence of impairments in mental functions over time.

	T0	T1	T2	T3
Brain fatigue	91.9	88.2	87.7	90.9
Memory problem	89.2	85.8	84.4	85.2
Concentration problem	87.3	88.3	86.3	86.2
Attention problem	86.8	86.2	87.3	85.7
Impaired mental functions of language	84.4	63.8	58.1	66.2
Impaired organization and planning	74.8	79.0	79.6	79.0
Sleep problems	73.7	79.7	80.9	80.9
Stress	69.5	74.9	74.3	76.6
Depression	59.4	62.7	54.2	58.1
Impaired orientation functions	58.9	54.6	46.9	48.3
Worry/anxiety	53.8	55.2	53.8	59.6
Lack of appetite	39.1	42.0	42.2	38.6
Obsessions	24.8	29.9	27.3	29.0
Compulsions	15.3	16.9	14.2	14.8

T0, baseline; T1, 3-month follow-up; T2, 6-month follow-up; T3, 12-month follow-up. The color intensity represents the frequency (expressed as a percentage) of individuals reporting symptoms. The symptoms are arranged in descending order, ranging from the least prevalent (blue) to the most prevalent (red).

Pain showed a general decrease over time. However, there were no changes observed in sensory functions during the one-year follow-up period (Table 5).

**Table 5:** Prevalence of impairments in sensory functions and pain over time.

	T0	T1	T2	T3
Pain in multiple body parts	80.1	61.3	64.9	59.3
Pain in head/headache	77.3	64.2	64.6	64.1
Dizziness	75.8	73.0	67.9	75.2
Feeling of numbness/tingling	72.5	66.0	69.7	67.5
Joint pain	72.0	66.0	67.3	63.6
Chest pain	68.6	53.1	52.2	53.8
Disturbed balance	66.7	69.5	68.9	75.2
Impaired quality of vision	61.6	61.3	61.6	59.9
Pain in stomach or abdomen	61.0	50.5	48.8	50.5
Ringing in ears or tinnitus	60.4	59.7	59.9	61.9
Dry/red/itchy eyes	59.9	58.5	62.3	61.0
Generalized pain	54.3	57.4	55.7	55.1
Loss of smell	44.3	37.9	43.3	45.7
Loss of taste	43.3	41.5	42.9	45.5

T0, baseline; T1, 3-month follow-up; T2, 6-month follow-up; T3, 12-month follow-up. The color intensity represents the frequency (expressed as a percentage) of individuals reporting symptoms. The symptoms are arranged in descending order, ranging from the least prevalent (blue) to the most prevalent (red).

Regarding impairments in body system functions, impaired mobility, decreased muscle power, and impaired heart functions exhibited notable decreases over time. Tiredness or lack of energy, along with shortness of breath, demonstrated a gradual yet significant decrease during the one-year follow-up period. On the contrary, respiratory distress displayed the most increase throughout this period (Table 6).

**Table 6:** Prevalence of impairments in body system functions over time.

	T0	T1	T2	T3
Tiredness or lack of energy (Fatigability)	95.7	92.5	92.9	91.9
Decreased muscle power	83.3	75.1	68.7	74.6
Shortness of breath	83.1	76.8	78.2	80.5
Impaired heart functions	82.4	56.9	56.0	59.1
Impaired mobility	68.9	36.3	37	35.9
Impaired sexual desire and functions	68.7	62.7	66.8	62.7
Weight change	65.1	67.6	60.4	66
Chills or the feeling of freezing	61.2	54.9	58.0	62.7
Fever/feeling of fever	56.7	56.3	56.7	56.7
Hair loss	55.3	48.8	48.3	50.5
Cough	54.3	41.0	44.4	47.1
Respiratory distress/Impaired respiratory functions	52.6	65.3	67.8	71.8
Nausea	51.2	46.2	50.5	48.8
Impaired control over and coordination of movements	50.2	51.7	47.4	51.5
Sore throat/difficult to swallow	45.4	37.0	40.9	41.4
Rash/itching	43.3	44.5	46.0	49.0
Voice problems	41.7	41.4	43.5	39.9
Skin changes	41.1	40.7	36.2	43.3
Diarrhea	39.2	34.0	39.4	38.3
Impaired nutrient uptake	33.0	35.7	36.5	38.8
Vomiting	15.6	11.8	13.9	12.4

T0: baseline; T1: 3-month follow-up; T2: 6-month follow-up; T3: 12-month follow-up. The color intensity represents the frequency (expressed as a percentage) of individuals reporting symptoms. The symptoms are arranged in descending order, ranging from the least prevalent (blue) to the most prevalent (red).

Furthermore, all symptoms related to impairments in activities and participation showed decline over time (Table 7).

**Table 7:** Prevalence of impairments in activities and participation over time.

	T0	T1	T2	T3
Difficulty participating in leisure activities	92.0	89.6	87.7	85.7
Impaired work ability/study ability	91.5	88.7	86.3	85.2
Difficulties in doing housework	74.3	67.5	64.9	68.4
Difficulty taking care of yourself	47.6	40.8	47.1	44.5
Impaired control of other diseases and drugs, keep special diet	38.2	21.7	28.8	33.5

T0, baseline; T1, 3-month follow-up; T2, 6-month follow-up; T3, 12-month follow-up. The color intensity represents the frequency (expressed as a percentage) of individuals reporting symptoms. The symptoms are arranged in descending order, ranging from the least prevalent (blue) to the most prevalent (red).

### 3.4 Factors related with changes in post-COVID impairments over time

We found a main effect of time for decreased impairments in mental functions, sensory functions and pain, and body system functions over time but not for impairments in activities and participation (Table 8). Additionally, individuals with lower levels of education and those who were not employed at baseline exhibited a heightened likelihood of experiencing a deterioration in post-COVID impairments over time. Receiving any type of treatment options for post-COVID complaints was associated with impairments in body system and impairments in activities and participation over time. Our findings further indicated that individuals initially infected with COVID-19 in 2020 and hospitalized due to the COVID-19 showed greater ongoing impairments in body system functions. The severity of COVID-19 infection during the acute phase was linked to higher odds of experiencing post-COVID impairments at any given time-point.

**Table 8:** The results from linear mixed models for predicting post-COVID impairments over time, adjusted for individuals.

	Impairments in mental functions	Impairments in sensory functions and pain	Impairments in body system functions	Impairments in activities and participation
	Estimate [95% CI]	Estimate [95% CI]	Estimate [95% CI]	Estimate [95% CI]
**Fixed effects**				
Intercept	0.92 ** [0.29, 1.55]	0.38 [−0.11, 0.88]	0.60 ** [0.18,1.01]	0.15 *** [0.79,2.19]
Time	−0.04 ** [−0.06, −0.01]	−0.04 ** [−0.06, −0.02]	−0.03 ** [−0.05,-0.01]	−0.03 [−0.06,−0.00]
Age (years)	-0.00 [−0.01,0.01]	0.00 [−0.00,0.01]	0.00 [−0.01,0.00]	−0.01 [−0.02,0.00]
Gender: female	−0.27 [−0.02,0.55]	0.08 [−0.14,0.31]	0.03 [−0.15,0.22]	0.09 [−0.23,0.40]
Education: lower education	0.22 * [−0.42,0.01]	0.29 ** [−0.45,-0.13]	0.22 ** [−0.35,-0.08]	0.15 [−0.38,0.08]
Marital status: not relationship	0.07 [−0.13,0.27]	0.06 [−0.10,0.22]	0.06 [−0.07,0.19]	0.06 [−0.16,0.28]
Work status: not working	0.12 [−0.32,0.09]	0.24 ** [−0.40,-0.08]	0.25 *** [−0.38, −0.12]	0.49*** [−0.72,-0.26]
Economic status: average and below average	0.15 [−0.33,0.03]	0.03 [−0.17,0.11]	0.01 [−0.13,0.10]	−0.03 [−0.18,0.23]
Vaccinated against COVID-19: vaccinated	−0.15 [−0.39,0.09]	−0.18 [−0.36,0.01]	−0.15 [−0.30,0.01]	−0.19 [−0.45,0.08]
Time of first infection: during year 2020	0.14 [−0.05,0.33]	0.15 [−0.00,0.30]	0.13 *[0.01,0.25]	0.22 [0.00,0.43]
Receiving treatment: received treatments	0.11 [−0.09,0.30]	0.16 [0.01,0.31]	0.18** [0.05,0.30]	0.44 ** [0.22,0.65]
Hospitalization for COVID-19: been hospitalized	0.17 [−0.07,0.42]	0.12 [−0.08–0.31]	0.20 * [0.04,0.36]	0.23 [−0.05,0.48]
Belong to risk group: been high-risk group	0.12 [−0.09,0.33]	0.08 [−0.09,0.24]	0.05 [−0.08,0.19]	0.13 [−0.11,0.36]
Severity of COVID-19 infection	0.41*** [0.23,0.60]	0.54 *** [0.40,0.68]	0.43 *** [0.31,0.55]	0.20 [−0.00,0.40]
**Random effects**				
Individual intercept	0.25	0.14	0.10	0.30
Residual	0.12	0.10	0.07	0.15
**Model fit indices**				
AIC	1.42	1.17	0.84	1.62
BIC	811.76	692.91	472.13	970.72

Coef, coefficient; CI, Confidence interval; AIC, Akaike Information Criterion; BIC, Bayesian Information Criterion. *p ≤ 0.05, **p < 0.01, ***p < 0.001. Bold values indicate a significant association.

## 4 Discussion

We aimed to investigate changes in post-COVID impairments over time and explore potential predictive variables influencing these changes in a longitudinal approach in a Swedish context. The results of this study revealed that post-COVID impairments tend to decrease across various categories over time. However, factors like the severity of the initial COVID-19 infection, education level, and work status played significant roles in the level of these impairments. The severity of the initial infection appeared to be a consistent predictor of higher impairment levels. Despite a general decline in post-COVID impairments, a distinct and dynamic pattern of fluctuations was observed for each symptom over time, indicating post-COVID impairments were not static but exhibited a range of variations over time.

### 4.1 Impartments in mental functions

The results revealed a persistent high level of impairments in mental functions even after one year. While a decline in trend was evident over time, the manifestation of the changes differed across various symptoms. Cognitive functions including orientation functions, mental functions of languages, concentration, and memory improved over time. Particularly, the improvements were notably more pronounced in orientation functions and mental functions related to language. The findings are consistent with previous studies showing the existence of structural and metabolic changes in the brain and the presence of autoantibodies in cerebrospinal fluid linked to cognitive impairment following COVID-19 infection (Hosp et al., 2021; Ferrucci et al., 2023; Franke et al., 2023). Furthermore, a comprehensive meta-analysis study demonstrated that cognitive impairment manifested in approximately one out of every four patients with post-COVID syndrome, persisting for three months or longer following the initial infection (Ceban et al., 2022). However, longitudinal studies that employed The Montreal Cognitive Assessment (MoCA) to evaluate cognitive functions reported post-infection symptoms established a decline in cognitive impairments, coinciding with an increase in the mean MoCA score over a one-year follow-up period (Liu et al., 2023; Steinmetz et al., 2023). Our findings also revealed that brain fatigue was still the most common symptom over time in line with other studies (Rass et al., 2022; Liu et al., 2023; Steinmetz et al., 2023). Nevertheless, neuropsychiatric symptoms like lack of appetite, depression, anxiety, stress, sleep problems, obsessions, and compulsions, along with higher-level cognitive functions such as organization and planning, exhibited only marginal changes, in some instances such as sleep problems a significant worsening trend was observed over time. These results are in line with our previous findings concerning the investigation of mental health changes in COVID-19-infected individuals, which revealed a decline in depression over time, while no significant changes were observed in anxiety and insomnia (Badinlou et al., 2024).

### 4.2 Impairments in sensory functions and pain

Impairments in sensory functions and pain displayed a general improvement over time. These improvements point toward the body’s capacity to overcome acute pain and discomfort. Pain emerges as a prevalent symptom among post-COVID patients, often interconnected with other symptoms and routine activities (Huang et al., 2022; Smith et al., 2023). Nonetheless, the findings regarding the temporal progression of pain are incongruent, with some studies reporting reductions (Steinmetz et al., 2023), while others indicating an increase over time (Huang et al., 2022). These differences may be due to methodological differences, encompassing variations in the post-COVID trajectory, and the timing of data collection. One possible explanation for the decrease in post-COVID pain may lie in the indication of the body’s ability to naturally heal and recuperate from the discomfort associated with the disease. These healing mechanisms activated in response to viral infections and inflammation underscore the gradual mitigation of pain (Chen et al., 2018). Another plausible explanation could involve the utilization of pain relief medications and pain management strategies adopted for post-COVID patients (Basu et al., 2022).

Regarding impairments in sensory functions, results revealed that sensory functions displayed minimal changes. The outcomes of a recent meta-analysis revealed that patients with COVID-19 exhibit statistically significant impairments in sensory functions such as hearing loss, tinnitus, and dizziness (Jafari et al., 2022). Other findings showed involvement of cranial nerves in COVID-19, resulting in the manifestation of sensory functions impairments (Doblan et al., 2021). However, an investigation into the olfactory, gustatory, hearing, and vestibular systems among post-COVID patients has brought to light a significant finding. In that, post-COVID patients experienced impairments in their sensory functions, and also reduced performance in balance tests due to problems to integrate vision, somatosensory information, and vestibular information months after COVID-19 infection, even after recovery from acute phase of the disease (Gervasoni et al., 2022; Ludwig et al., 2022).

### 4.3 Impairments in body system functions

Our findings revealed that impairments in body system functions exhibit a significant reduction over time. Fatigue and muscle weakness consistently emerged as prevalent impairments in body system function. The results are in line with prior research findings spotlighting fatigue and muscle weakness as the most prominent symptoms months after infection, regardless of initial disease severity (Lopez-Leon et al., 2021; Dos Santos et al., 2022; Soares et al., 2022). However, a fluctuating pattern of changes was observed when considering each symptom.

Our findings revealed that neuromusculoskeletal and movement-related functions including mobility and muscle power improved notably. Extensive documentation supports the fact that the musculoskeletal system is impacted by COVID-19, even following mild to moderate infections (Dos Santos et al., 2022). However, the longitudinal study showed a significant recovery in muscle strength and mobility between 3- and 12-months following discharge from hospital (Lorent et al., 2022). These findings suggest that the musculoskeletal system might require over a year for a recovery from the effects of COVID-19.

In our study the most notable improvement was observed in impaired heart functions. Impairments in functions of the cardiovascular system were observed widely in mild to severe acute COVID-19 as well as in post-COVID patients (Skaarup et al., 2020; Lassen et al., 2021; Singh et al., 2022). However, longitudinal studies highlight those certain cardiovascular manifestations showed notable improvement over time, while others did not improve remarkably, depending on factors like time since acute COVID-19 infection, the patient population, and pre-existing cardiovascular abnormalities (Joy et al., 2021; Lassen et al., 2021; Gyöngyösi et al., 2023). Despite the variability in recovery times for different cardiovascular issues attributed to COVID-19, spanning cellular to clinical features, this study underscores that individuals subjectively experience significant recovery over time. Contrary to the reported improvement in heart problems, individuals in the current study reported an increase impairment in their respiratory functions over the course of the study. Given that SARS-CoV-2 primarily affects the respiratory system, our findings align with previous research indicating that impairments in pulmonary function persist for months after hospital discharge (Krueger et al., 2023). However, our results are inconsistent with previous studies (Tarraso et al., 2022; Schlemmer et al., 2023) reporting an improvement in pulmonary function over time. The first possible explanation for this finding lies in methodological differences. The respiratory functions are predominantly assessed through objective clinical tests by healthcare professionals in healthcare systems. In contrast, participants in our study were asked to subjectively rate their respiratory problems. This shift in assessment methods, from objective clinical tests to subjective participant ratings, may contribute to the observed differences. A second possible explanation for the observed differences in our study compared to previous research lies in the characteristics of the study population. Previous research primarily focused on patients discharged from hospitals. In contrast, a majority of participants in our study experienced mild COVID-19 infections during the acute phase of the disease. It can be concluded that hospitalized patients might represent a subset with more severe cases, potentially undergoing different recovery trajectories than individuals with milder forms of the disease. A third possible explanation is the timeframe of data collection, where discrepancies in the duration of follow-up periods between studies may influence the observed trends. A forth possible explanation is the formulation of the related survey question. The utilization of a single question, aimed at encompassing a broad spectrum of potential respiratory issues, may have inadvertently led to an increased reported rate of experienced impaired respiratory function.

Functions related to the digestive system exhibited marginal decreases over time. Meta-analyses have consistently highlighted the relatively common occurrence of impairments in the digestive system during the acute phase of COVID-19 infection (Cheung et al., 2020; Segura et al., 2020; Wang et al., 2021). The intricate relationship between COVID-19 and the digestive system lies in the fact that SARS-CoV-2 can actively infect and replicate within the gastrointestinal tract, leading to damage and abnormalities in the digestive system (Ma et al., 2020, 2022; Wong et al., 2020; Cao et al., 2021). However, the prevalence of gastrointestinal symptoms and the underlying pathophysiology of potential gut infections in patients with post-acute sequelae of SARS-CoV-2 remain subjects of investigation (Anaya et al., 2021; Silva Andrade et al., 2021; Norouzi Masir and Shirvaliloo, 2022). Additional findings have also indicated that the effects of pandemic-related stress may contribute to gastrointestinal symptom manifestation and perception (Freedberg and Chang, 2022). Nevertheless, longitudinal studies have demonstrated a decline in symptoms over time as observed in the current study (Freedberg and Chang, 2022). These findings highlight that the multifaceted nature of gastrointestinal symptoms and their progression in not only acute but also post-acute phases of the disease.

Functions related to metabolism and the endocrine system exhibited marginal changes over time. A meta-analysis by Islam et al. found that 79.43% of individuals infected with COVID-19 experienced fever, mainly medium-grade fever, and 14.45% of adult COVID-19 patients were reported to have chills as a symptom (Islam et al., 2021). However, both fever and chills, present in the acute stage of infection, resolved in most individuals over time (Raveendran et al., 2021). The prevalence of impairments in the metabolism and endocrine system, as evidenced in the current study, was high at one-year follow-up. One potential explanation for these findings could be attributed to the formulation of the related survey questions. Participants in the present study were asked to rate their experience of fever or/and feeling of fever as well as chills and/or feeling of freezing. In other words, it might be concluded that participants perceived feelings of fever and freezing even without actual fever and chills being present.

### 4.4 Impairments in activities and participation

Our findings revealed a declining trend in impairments associated with activities and participation, highlighting a significant improvement in the ability to work and study. There are several explanations for these findings. Firstly, a substantial proportion of our participants belonged to a working population, and the disruptions induced by the pandemic and post-COVID complications may have adversely affected their work and study routines. This aligns with our previous study, which, based on baseline data, indicated that 87.3% of participants identified impaired work or study ability as the most affected aspect of their lives (Badinlou et al., 2023). This observation implies that there is considerable potential for enhancement in work and study ability as time progresses in our sample, in line with a previous study (Abzhandadze et al., 2024). Secondly, a significant number of participants experienced mild COVID-19 infections, potentially leading to shorter recovery times. This factor could contribute to the observed positive trajectory in impairments related to work and study abilities. Thirdly, the interventions and support initiatives seem to play a crucial role in facilitating a positive impact on participants’ ability to return to work and engage in their studies. This multifaceted approach, combining occupational considerations, the nature of the COVID-19 infections and post-COVID complications, and supportive interventions, contributes to a comprehensive understanding of the observed trends.

### 4.5 Factors related with changes in post-COVID impairments over time

The mixed model analysis revealed that sociodemographic factors, including education and working status, were associated with post-COVID impairments over time, consistent with previous studies (Abdelghani et al., 2022; Müller et al., 2023), in that individuals with higher educational levels and those employed at the baseline were more likely to experience a decline in post-COVID impairments over time. Several possible explanations may account for these findings. Firstly, individuals with lower education levels were less likely to adhere to COVID-19 preventive measures, creating challenges or barriers that impede their ability to follow recommended precautions and elevating their risk of contracting COVID-19 infection (Hassan et al., 2020; Abeya et al., 2021; Folayan et al., 2023). Secondly, educational attainment has been identified as a predictor of hospitalization due to COVID-19 infection and disease severity during the acute phase (Jian et al., 2021). Thirdly, education level has emerged as a key factor in shaping attitudes toward the COVID-19 vaccine and willingness to be vaccinated (Hudson and Montelpare, 2021; Lazarus et al., 2021). Fourth, individuals with lower education levels are at higher risk of experiencing post-COVID symptoms (Bovil et al., 2023). These findings revealed the association between education level and health literacy (Van der Heide et al., 2013), highlighting the multifaceted role of education in shaping individuals’ behaviors, perceptions, and health-related decision-making processes in the context of a global health crisis such as the COVID-19 pandemic. It is therefore reasonable to conclude that educational disparities can influence not only susceptibility to the virus but also the subsequent health outcomes.

More findings revealed the association between unemployment status and the persistence of post-COVID impairments over time. Our previous findings strongly indicate that being unemployed is a robust predictor for post-COVID impairments, particularly in activities and participation (Badinlou et al., 2023). These findings are consistent with previous studies highlighting that the post-COVID conditions is linked to a higher likelihood of unemployment and a reduced probability of full-time employment (Perlis et al., 2023). Extensive documentation supports the notion that post-COVID complications are associated with diminished working ability and/or subsequent occupational changes, due to both physical and mental demands (Davis et al., 2021; Heightman et al., 2021; Kisiel et al., 2022; Kerksieck et al., 2023; Miskowiak et al., 2023; Van Wambeke et al., 2023; Lunt et al., 2024).

Our results revealed a significant and positive association between the severity of COVID-19 infection in the acute phase and the emergence of post-COVID impairments over time. More specifically, severity of COVID-19 infection in the acute phase emerged as the most robust predictor for impairments in various domains, including mental functions, sensory functions and pain, and body system functions. However, it is crucial to acknowledge that there are inconsistencies in research findings concerning the relationship between the severity of the disease during the acute phase and the persistence of symptoms. While some investigations, including our study, support the notion that initial severity is a robust predictor of enduring impairments over time (Huang et al., 2021; Truffaut et al., 2021; Weerahandi et al., 2021; Zhang et al., 2021; Han et al., 2022; Badinlou et al., 2023), other research suggests there is no clear link between acute disease severity and the persistence of symptoms (Townsend et al., 2020; Bell et al., 2021; Moreno-Pérez et al., 2021; Yong, 2021; Ko et al., 2022; Pérez-González et al., 2022; Stephens et al., 2024). These disparities in research outcomes highlight the intricate nature of post-COVID-19 trajectories.

## 5 Strengths and limitations

The current study is subject to several limitations. Primarily, the dependence on self-reported data to assess COVID-19 impairments introduces risks of recall and social desirability biases, as well as the subjective interpretation of symptoms. Such a methodology may not achieve the accuracy and validity characteristic of clinical assessments. Additionally, the employment of a convenience sample in this study limits the generalizability of the findings, underscoring the necessity for subsequent research to mitigate potential biases in sample selection and to ensure a more representative cohort.

A further concern is the possibility of non-response bias, as participants who remain in subsequent phases of the study may exhibit characteristics differing from those who withdrew, potentially skewing the results and affecting the representativeness of the sample. Additionally, the absence of a control group unaffected by COVID-19 complicates the delineation of the specific impacts of the infection on post-COVID-19 impairments.

The four-wave longitudinal design of the study may not sufficiently capture the long-term effects of COVID-19. This limitation indicates the requirement for more comprehensive longitudinal studies with additional data collection phases to enhance understanding of these impacts over an extended period. Furthermore, external and contextual factors, such as social support, access to rehabilitation services, and the overall societal response to the pandemic, may have influenced the observed changes in post-COVID-19 impairments. Therefore, further research is imperative to identify these underlying factors and to develop targeted interventions.

Moreover, the characteristics of the study sample could potentially limit the generalizability of the findings. For instance, the sample consisted of individuals living in Sweden and the majority of the participants in the current study were female, with higher education levels, married, employed, reported average to above-average economic status, were vaccinated against COVID-19, and were non-hospitalized. Acknowledging these characteristics is crucial because they may impact the applicability of the study findings to broader populations.

In interpreting these findings, it is crucial to consider these limitations and the potential for confounding variables. Although the study provides insightful observations regarding the trajectories of post-COVID-19 impairments, more extensive research is vital to elucidate the factors contributing to these outcomes and to formulate specialized interventions for individuals experiencing prolonged impairments post-infection.

## 6 Conclusion

This study on post-COVID impairments revealed a general trend of decreasing prevalence over time. However, it also found that individuals with lower education levels and those who are unemployed were more likely to experience worsening conditions, highlighting the significant influence of educational background and employment status on the trajectory of post-COVID impairments. The severity of the initial COVID-19 infection emerged as a key factor in ongoing impairments. These findings underscore the necessity for personalized care strategies, especially for vulnerable populations. Clinically, this translates into the need for long-term monitoring and multidisciplinary treatment approaches. The results highlight the importance of targeted support and interventions for those with severe initial infections and those in socioeconomically disadvantaged groups.

## Data availability statement

The raw data supporting the conclusions of this article will be made available by the authors, without undue reservation.

## Ethics statement

The study was approved by the Swedish national ethical board (dnr 2021-06617-01). The studies were conducted in accordance with the local legislation and institutional requirements. The participants provided their written informed consent to participate in this study.

## Author contributions

FB: Conceptualization, Data curation, Formal analysis, Funding acquisition, Investigation, Methodology, Project administration, Resources, Software, Supervision, Validation, Visualization, Writing – original draft, Writing – review & editing. TA: Validation, Visualization, Writing – review & editing, Writing – original draft. FR: Formal analysis, Software, Writing – review & editing, Methodology. MJ-F: Conceptualization, Investigation, Methodology, Supervision, Validation, Visualization, Writing – review & editing. MH-L: Visualization, Writing – review & editing. TL: Conceptualization, Investigation, Methodology, Supervision, Validation, Visualization, Writing – review & editing.
