# Supplementary material for: Investigating the trajectory of post-COVID impairments: a longitudinal study in Sweden
Source: Front Psychol. 2024 Jun 10;15:1402750. doi: 10.3389/fpsyg.2024.1402750 (PMC11195806; doi:10.3389/fpsyg.2024.1402750)
Supplement: Supplementary file 1 [file Table_1.docx]

**Investigating the Trajectory of Post-COVID Impairments: A Longitudinal Study in Sweden**

[Farzaneh Badinlou](https://www.nature.com/articles/s41598-023-33810-y#auth-Farzaneh-Badinlou)^1,2†^*, Tamar Abzhandadze^3,4,5^ Fatemeh Rahimian^6^, [Markus Jansson-Fröjmark](https://www.nature.com/articles/s41598-023-33810-y#auth-Markus-Jansson_Fr_jmark)^1^, Maria Hedman-Lagerlöf^1^, Tobias Lundgren^1†^

1. Centre for Psychiatry Research, Department of Clinical Neuroscience, Karolinska Institute & Stockholm Health Care Services, Region Stockholm, Stockholm, Sweden

2. Medical Unit Allied Health Professionals, Women’s Health and Allied Health Professionals Theme, Karolinska University Hospital, Solna, Sweden

3. Institute of Neuroscience and Physiology, The Sahlgrenska Academy, University of Gothenburg, Gothenburg, Sweden

4. Department of Occupational Therapy and Physiotherapy, Sahlgrenska University Hospital, Gothenburg, Sweden

5. Division of Clinical Geriatrics, Department of Neurobiology, Care Sciences and Society (NVS), Karolinska Institutet, Stockholm, Sweden

6. RISE Research Institutes of Sweden, Department of Computer Science, Stockholm, Sweden

^†^ These authors share last authorship

*  **Correspondence:**

Dr Farzaneh Badinlou

Farzaneh.badinlou@ki.se

**Supplementary Table 1.** Items of post-COVID impairments according to ICF and descriptive statistics at the four time points (mean/SD)

| ICF code | Item | T0 | T1 | T2 | T3 | Difference T3-T0 |
| --- | --- | --- | --- | --- | --- | --- |
| Impairments in mental functions | |  |  |  |  |  |
| b114 | Impaired orientation functions such as difficulty of knowing and ascertaining one’s relation to self, to others, to time and to one’s surroundings | 1.05  (1.04) | .90  (.97) | .79  (.96) | .75  (.89) | -.3 *** |
| b 1300 | Brain fatigue | 2.21  (.90) | 2.04  (.97) | 1.96  (.98) | 2.06  (.96) | -.15 ** |
| b 1302 | Lack of appetite | .71  (1.07) | 0.69  (.91) | .70  (.92) | .64  (.90) | -.07 |
| b 134 | Sleep problems | 1.48  (1.13) | 1.66  (1.05) | 1.65  (1.07) | 1.65  (1.06) | .17 * |
| b 140 | Concentration problem such as difficulty of focusing on a task or topic | 1.97  (1.08) | 1.93  (.98) | 1.83  (1.01) | 1.90  (1.03) | -.07 |
| b 140 | Attention problem such as difficulty of focusing on one part of an experience and ignoring others or focusing on several things | 1.99  (1.02) | 1.99  (1.04) | 1.99  (1.02) | 2.03  (1.08) | .04 |
| b 144 | Memory problem | 1.91  (.96) | 1.77  (.98) | 1.75  (.99) | 1.76  (.98) | -.15 ** |
| b 1641 | Impaired organization and planning such as difficulty of coordinating parts into a whole) | 1.67  (1.15) | 1.67  (1.08) | 1.68  (1.07) | 1.68  (1.08) | -.01 |
| b 167 | Impaired mental functions of language such as difficulty of recognizing and using signs, symbols and other components of a language | 1.68  (1.01) | 1.14  (1.03) | .94  (.95) | 1.14  (1.01) | -.54 *** |
| b 152 | Depression | .98  (.96) | 1.02  (.95) | .87  (.93) | .96  (.98) | -.02 |
| b 152 | Worry/ anxiety | .92  (1.04) | .94  (1) | .90  (.99) | 1  (.98) | .08 |
| b 152 | Stress | 1.38  (1.11) | 1.41  (1.04) | 1.38  (1.05) | 1.42  (1.01) | .04 |
| b 160 | Obsessions such as unwanted thoughts and fears | .35  (.70) | .46  (.80) | .44  (.80) | .44  (.79) | .09 |
| b 160 | Compulsions such as you feel compelled to do certain things | 0.17  (.42) | .23  (.59) | .20  (.57) | .20  (.57) | .03 |
| Impairments in sensory functions and pain | |  |  |  |  |  |
| b 2102 | Impaired quality of vision | 1.04  (1) | 1.01  (.97) | .99  (.94) | .98  (.97) | -.06 |
| b 220 | Dry / red / itchy eyes | 1.08  (1.06) | .97  (.99) | 1.08  (1.04) | 1.09  (1.06) | .01 |
| b 2400 | Ringing in ears or tinnitus | 1.15  (1.12) | 1.19  (1.16) | 1.16  (1.13) | 1.20  (1.13) | .05 |
| b 2401 | Dizziness | 1.26  (.93) | 1.26  (.99) | 1.14  (1) | 1.25  (.97) | -.01 |
| b 235 | Disturbed balance | 1.19  (1.03) | 1.19  (1) | 1.14  (.99) | 1.22  (.95) | .03 |
| b 250 | Loss of taste | .71  (.96) | .70  (.97) | .64  (.86) | .74  (.95) | .03 |
| b 255 | Loss of smell | 0.77  (1) | .67  (1) | .67  (.89) | .78  (.99) | .01 |
| b 265 | Feeling of numbness / tingling | 1.30  (1.01) | 1.16¨  (1.03) | 1.18  (.99) | 1.22  (1.06) | -.08 |
| b 2800 | Generalized pain | 0.99  (1.06) | 1.01  (1.04) | 1.04  (1.04) | 1.09  (1.15) | .1 |
| b 28010 | Pain in head | 1.49  (1.04) | 1.18  (1.06) | 1.18  (1.05) | 1.25  (1.24) | -.24 ** |
| b 28011 | Chest pain | 1.25  (1.04) | .94  (1.03) | .87  (.99) | .93  (1) | -.32 *** |
| b 28012 | Pain in stomach or abdomen | 1.13  (1.06) | .79  (.92) | .83  (.97) | .89  (1.02) | -.24** |
| b 28016 | Joint pain | 1.29  (1.02) | 1.16  (1.05) | 1.18  (1.05) | 1.23  (1.1) | -.06 |
| b 2802 | Pain in multiple body parts | 1.55  (1.04) | 1.10  (1.06) | 1.16  (1.05) | 1.13  (1.1) | -.42 *** |
| Impairments in body system functions | |  |  |  |  |  |
| b 310 | Voice problems | .71  (.97) | .65  (.89) | .67  (.88) | .62  (.88) | -.09 |
| b 410 | Impaired heart functions | 1.73  (1.07) | 1.06  (1.1) | .99  (1.04) | 1.04  (1.06) | -.69 *** |
| b 440 | Respiratory distress (Impaired respiratory functions) | .95  (1.04) | 1.21  (1.08) | 1.19  (1.02) | 1.28  (1.02) | .33 *** |
| b 450 | Cough | .86  (.95) | .65  (.93) | .71  (.93) | .73  (.92) | -.13 |
| b 4552 | Tiredness or lack of energy (Fatiguability) | 2.40  (.78) | 2.28  (.95) | 2.25  (.92) | 2.29  (.93) | -.11 * |
| b 460 | Shortness of breath | 1.69  (1.07) | 1.47  (1.08) | 1.41  (.99) | 1.57  (1.03) | -.12* |
| b 5105 | Sore throat/ difficult to swallow | .66  (.84) | .57  (.86) | .61  (.84) | .61  (.86) | -.05 |
| b 5106 | Vomiting | .25  (.65) | .16  (.5) | .21  (.59) | .18  (.56) | -.07 |
| b 515 | Impaired nutrient uptake | .61  (.97) | .51  (.78) | .54  (.82) | .59  (.87) | -.02 |
| b 525 | Diarrhoea | .63  (.90) | .54  (.85) | .63  (.90) | .58  (.84) | -.05 |
| b 530 | Weight change | 1.31  (1.14) | 1.28  (1.1) | 1.17  (1.13) | 1.27  (1.11) | -.04 |
| b 5350 | Nausea | .81  (.94) | .68  (.87) | .76  (.88) | .72  (.87) | -.09 |
| b 5500 | Fever/ feeling of fever | .95  (.98) | .98  (1.04) | .95  (1) | 1.03  (1.06) | .08 |
| b 5500 | Chills or the feeling of freezing | 1.16  (1.08) | .92  (1.02) | 0.99  (1.03) | 1.12  (1.07) | -.04 |
| b 640 | Impaired sexual desire and functions | 1.42  (1.17) | 1.16  (1.08) | 1.31  (1.14) | 1.31  (1.21) | -.11 |
| b 710 | Impaired mobility such as difficulty to move knee, ankle, hands, feet. | 1.30  (1.07) | .58  (.91) | .63  (.94) | .66  (1.01) | -.64 *** |
| b 730 | Decreased muscle power | 1.70  (1) | 1.3  (.99) | 1.18  (1.01) | 1.34  (1.02) | -.36 *** |
| b 760 | Impaired control over and coordination of movements | .82  (.99) | .79  (.91) | .78  (.98) | .81  (.97) | -.01 |
| b 810 | Skin changes | .63  (.86) | .69  (.96) | .58  (.88) | .67  (.91) | .04 |
| b 840 | Rash/ itching | .71  (.93) | .71  (.93) | .72  (.90) | .79  (.96) | .08 |
| b 850 | Hair loss | .98  (1.03) | .90  (1.06) | .87  (1.06) | .93  (1.08) | -.05 |
| Impairments in activities and participation | |  |  |  |  |  |
| d 5 | Difficulty taking care of yourself | .82  (.99) | .69  (.94) | .76  (.93) | .73  (.94) | -.09 |
| d 5 | Impaired control of other diseases and drugs, keep special diet | .65  (.94) | .41  (.86) | .47  (.85) | .57  (.91) | -.08 |
| d 6 | Difficulties in doing housework | 1.42  (1.07) | 1.39  (1.18) | 1.32  (1.17) | 1.41  (1.16) | -.01 |
| d 8 | Impaired work ability / study ability | 2.25  (.97) | 2.02  (1.06) | 1.96  (1.08) | 2.02  (1.11) | -.23 *** |
| d 9 | Difficulty being to leisure activities | 2.24  (.97) | 2.14  (1.04) | 2  (1.09) | 2.14  (1.11) | -.1 |

T0: baseline; T1: 3-month follow-up; T2: 6-month follow-up; T3: 12-month follow-up

**p* < .05, ***p* < .001, ****p* < .000.
